# Parent and child perceptions of school-based obesity prevention in England: a qualitative study

**DOI:** 10.1186/s12889-015-2567-7

**Published:** 2015-12-09

**Authors:** Joanne L. Clarke, Tania L. Griffin, Emma R. Lancashire, Peymane Adab, Jayne M. Parry, Miranda J. Pallan, on behalf of the WAVES study trial investigators

**Affiliations:** Institute of Applied Health Research, Public Health building, University of Birmingham, Edgbaston, Birmingham, B15 2TT UK

**Keywords:** Child obesity, Stakeholder views, Primary school, Healthy eating, Physical activity, Focus groups, Process evaluation, Intervention

## Abstract

**Background:**

Schools are key settings for childhood obesity prevention, and the location for many intervention studies. This qualitative study aims to explore parent and child experiences of the WAVES study obesity prevention intervention, in order to gain understanding of the mechanisms by which the intervention results in behaviour change, and provide context to support interpretation of the main trial results.

**Methods:**

Focus groups were held with 30 parents and 62 children (aged 6-7 years) from primary schools in the West Midlands, UK. Data analysis (conducted using NVivo 10) was guided by the Framework Approach.

**Results:**

Three over-arching themes were identified: ‘Impact’, ‘Sustainability’ and ‘Responsibilities’, under which sub-themes were determined. Participants were supportive of the school-based intervention. Parental involvement and the influential role of the teacher were seen as key ingredients for success in promoting consistent messages and empowering some parents to make positive behavioural changes at home. Parents recognised that whilst they held the primary responsibility for obesity prevention in their children, they faced a number of barriers to healthier lifestyles, and agreed that schools have an important role to play.

**Conclusions:**

This study enabled us to better understand aspects of the WAVES study intervention programme that have the potential to initiate positive behaviour changes in families, and indicated that a combination of pathways influenced such changes. Pathways included: increasing capability through improving knowledge and skills of children and parents; increasing motivation through parental empowerment and role modelling; and the direct provision of opportunities to lead healthier lifestyles. Strategies to sustain behaviour changes, and the school role in supporting these, are important considerations.

## Background

Childhood obesity is a global public health challenge [1], and its health consequences are well-documented [2]. Schools offer an environment in which eating and activity occur, providing opportunities to learn about and implement healthy behaviours. In addition, they have the potential to engage parents to support activities in the home setting [3, 4], and promote consistent messages between home and school. Schools are therefore often seen as an important setting for childhood obesity prevention interventions [5].

Systematic review evidence indicates that school-based obesity prevention programmes targeting both physical activity and eating behaviours can be effective [3]. The complexity and heterogeneity of such interventions, however, make it difficult to disentangle the relative effectiveness of individual components and their potential interactions [6]. Qualitative techniques can be useful in generating data which provide insight into the attitudes, perceptions, motivations, concerns and opinions of participants [7]. This in turn helps us to understand and contextualise the active ingredients, and their mechanism of action, within interventions [8].

The West Midlands ActiVe lifestyle and healthy Eating in School children (WAVES) study is an ongoing cluster randomised controlled trial evaluating the effectiveness of an obesity prevention intervention for children aged 6-7 years. From 54 randomly selected primary schools in the West Midlands, UK, 26 were allocated at random to the intervention arm of the trial. For logistical reasons, half of the schools were scheduled to receive the 12-month intervention in 2011-12, the remainder in 2012-13. Full details of the WAVES study are described elsewhere [9], but in summary, the intervention focused on promoting healthy eating and physical activity. Teachers were asked to: (i) Incorporate an extra 30 min of physical activity into each school day; (ii) Deliver three cooking workshops with children and parents focusing on nutrition education and food preparation skills; (iii) Supervise class attendance at Villa Vitality, a healthy lifestyle programme run at an English Premier League football club; (iv) Distribute two signposting sheets with ideas on how to be more active and specifically directing families to local physical activity opportunities, and a termly newsletter to reiterate the importance of healthy lifestyles.

This qualitative study aims to explore parent and child experiences of the WAVES study, in order to gain understanding of the mechanisms by which the intervention results in behaviour change, and provide context to support interpretation of the main trial results. Although a number of studies have investigated parent and child views in the development phase of obesity prevention interventions [10–14], there is a paucity of published research on their views in the evaluation phase of such interventions. In addition, recent guidance emphasises the importance of considering and presenting qualitative findings ahead of the main trial outcome to minimise interpretation bias [15]. This qualitative study was conducted as part of the WAVES study process evaluation [16]; related findings from interviews with teachers have previously been reported [17].

## Methods

This study uses a descriptive-interpretive qualitative methodology [18]. A sub-sample of schools participating in the WAVES study intervention programme was purposively selected to ensure contributions from a range of schools (diverse in location, ethnic mix of pupils, school size and deprivation (indicated by free school meal entitlement)). Data collection took place towards the end of the intervention period (May-July 2012 or May-July 2013). Ethical approval was obtained from the National Research Ethics Service Committee West Midlands, The Black Country (10/H1202/69). Parents provided written consent for themselves and/or their child prior to the focus groups. A £5 shopping voucher was given to parents attending the focus groups.

Ten schools (out of 15 invited) agreed to participate in this qualitative study. Three schools declined due to time pressures and the remaining two failed to respond. In the 10 participating schools, teachers were given letters of invitation to distribute to the parents of all children in their class (380 letters in total) inviting them to take part and/or permit their child to take part in a WAVES study focus group. Two of the schools held child focus groups but advised against the running of parent focus groups in their schools due to an anticipated poor response from parents. One school held a parent focus group but was unable to hold a child focus group due to time constraints in the curriculum. In total, 30 parents and 62 children participated in the study. Seven parent focus groups (mean group size, n = 4; range 2-12) (plus one interview (n = 1) because only one parent attended a planned focus group), and 13 child focus groups (mean group size, n = 5; range 2-7) were conducted. Characteristics of the schools, and participant numbers, are shown in Table 1.

**Table 1:** Characteristics of schools involved in the focus group study, and number of participants

School number	Year of intervention	School size (no. on roll)	Free school meal eligibility (%)	Ethnicity (% white)	Participants	
					Parents	Children
1	2011/12	<200	10-19	20-29	2 mothers	2 girls, 7 boys
2	2011/12	<200	20-39	90-99	-	4 girls, 3 boys
3	2011/12	≥300	40-60	50-59	1 mother	3 girls, 5 boys
4	2011/12	200-299	20-39	60-69	-	4 girls, 2 boys
5	2012/13	200-299	20-39	0-9	4 mothers	2 girls, 3 boys
6	2012/13	≥300	40-60	20-29	2 mothers, 1 father	6 girls, 4 boys
7	2012/13	≥300	20-39	60-69	2 mothers, 1 father	4 girls, 3 boys
8	2012/13	<200	0-9	90-99	2 mothers, 1 father	-
9	2012/13	≥300	10-19	70-79	2 mothers	3 boys
10	2012/13	200-299	10-19	10-19	10 mothers, 2 fathers	3 girls, 4 boys
Total					25 mothers, 5 fathers	28 girls, 34 boys

Focus groups were run by two female researchers with training in qualitative research methods (J Clarke, MSc, Research Associate, and T Griffin, PhD, Research Fellow). One researcher led the focus group, whilst the other made field notes (contextual details and non-verbal expressions to aid data analysis and interpretation). The researchers were previously known by some participants through school visits as part of the WAVES study. Child and parent focus groups were conducted separately, within the participants’ school, without the presence of school staff (except in one child focus group where a teaching assistant helped a child with additional needs, but made no contribution to the discussion). A 45-min time slot was made available for each focus group. Average duration of discussion was 24 min for children and 28 min for parents. Topic guides (Table 2) were used to help direct discussions and participants were encouraged to talk openly about their experiences. Within parent and child focus groups, participants were asked to recount their experience of the WAVES study overall, and of the separate intervention components (additional physical activity, cooking workshops, Villa Vitality, signposting). Parents were also asked to consider any beneficial effects of the intervention (including any behaviour change) as well as the wider role of the school in preventing obesity.

**Table 2:** Topic Guides for parent and child focus groups, to explore experiences of school-based obesity prevention

Topic guide: Parent focus groups
1: Can you tell me what you know about the WAVES study and the activities it involved?
2: Can you tell me about you and your child’s overall experience of being involved in the WAVES study?
3: As part of the WAVES study programme, schools were asked to fit in an extra 30 minutes of activity into the school day. Did you know this was happening in your child’s school? How do you feel about it?
4: What did you think about the signposting sheets?
5: What did you think of the cooking workshops? Do you think the workshops had any impact on your family?
6: Your child’s class also attended Aston Villa football club for the Villa Vitality programme. What do you think your child’s experience of the Villa Vitality programme was?
7: Do you think there were components of the WAVES study programme which were more beneficial than others?
8: Do you think the WAVES study programme of activities had any effect on your child’s behaviours and attitudes towards healthy lifestyle behaviours?
9: What effect (if any) do you think the WAVES study programme has had on your family’s lifestyle habits?
10: What role (if any) do you think schools play in obesity prevention?
Topic guide: Child focus groups
1: Can you tell us what you know about the WAVES Study? What did you do as part of the WAVES study?
2: What did you think of the cooking workshops in school? Did you learn anything new?
3: Can you tell me what you think about the WAVES study physical activities? How do they make you feel?
4: What did you think about the Villa Vitality programme? What did you do at Villa Vitality?
5: Did you take part in the Villa Vitality challenges? What did you think of the challenges?

Due to the young age of the children in this study (6-7 years), the facilitation of focus groups required special attention. As recommended by Stewart and Shamdasani [19], the moderators (JC and TG) were experienced in working with young children. First names were used to moderate the hierarchical adult-child relationship [20], and a short, fun ice-breaker helped children to feel comfortable and relaxed. Discussion was encouraged through the use of photographs of the intervention activities, and further prompts were used when necessary to clarify children’s responses.

Group discussions were voice recorded, transcribed verbatim, and anonymised. Thematic data analysis, guided by the Framework Approach [21], was undertaken in five stages: data familiarisation, theme identification, indexing, charting, and mapping the data. As recommended by Gale et al., [22], two researchers (JC and TG) independently reviewed all transcripts, identified themes and applied codes to the data. Codes were compared and discussed, and a thematic framework agreed. This framework was applied (independently) to the transcripts which were indexed using NVivo 10 (QSR International Pty Ltd. Version 10, 2012). At first, child and parent data were analysed separately, but due to the identification of common themes, the two datasets were subsequently reviewed together by all authors to identify and map overarching themes. For pragmatic reasons, member checking was not implemented.

## Results

Three overarching themes were identified from the data: ‘Impact (of the WAVES study)’, ‘Sustainability’ and ‘Responsibilities (for obesity prevention)’, under which sub-themes were determined. The ‘Impact’ and ‘Responsibilities’ overarching themes, and the ‘role of schools’ subtheme arose from the Topic Guide, and thus were researcher-led. All other themes emerged from the data analysis. Fewer themes were generated from the focus group discussions with children than with parents, and these were mainly assigned to ‘Impact’. Table 3 shows all themes, and indicates whether these arose from both parent and child discussions, or just from the parent discussions.

**Table 3:** Themes identified from focus group discussions exploring experiences of school-based obesity prevention

Overarching theme	Sub-theme	Discussed by parents and/or children
Impact (of the WAVES study)^a^	Improved knowledge and skills among children and parents	Parents and children
	Children trying new foods	Parents and children
	Implementing changes in the home	Parents
	Parental empowerment	Parents
	Role modelling	Parents and children
	Children as agents of change in the home	Parents
Sustainability	Sustainability of messages	Parents
	Sustainability of school-based programmes	Parents
Responsibilities (for obesity prevention)^a^	Role of parents	Parents
	Role of schools^a^	Parents and children
	Schools in partnership with parents	Parents

^a^Themes arising from topic guides; all other themes ‘emerged’ from the data

### Impact (of the WAVES study obesity prevention intervention)

#### Improved knowledge and skills among children and parents

It was evident that children could recall key messages from the WAVES study intervention programme, and were enthusiastic in sharing their knowledge within the focus group discussions; *‘fibre gives you an energy boost and it gives you energy for longer not like sugars, the sugars just give you energy for one minute’* (School 5, child). Children also displayed an understanding of the importance of healthy lifestyles; *‘if you don’t eat a healthy breakfast every morning then when you go to school you won’t be able to learn, you’ll go to sleep or something’* (School 5, child).

Following the intervention, participants reported that their interest in food preparation had increased. Children were particularly proud of their new skills (for example, in the safe use of knives to chop vegetables). They were equally keen to demonstrate their learning and practise their skills within the home environment, as one child explained; *‘I teached my mum how to cook it when we cooked in Aston Villa. And I chop a bit at home because I learned how to chop at Aston Villa’* (School 10, child).

Alongside reports of improved knowledge of children, a number of parents also reported that their own knowledge had improved as a result of the intervention; *‘I think it’s educated us as a parent a lot’* (School 10, mother). For others, the intervention served more as a reminder, with some parents intimating that although they already possessed the knowledge required to lead a healthy lifestyle, the intervention helped them think about, and possibly refine, their family health behaviours; *‘it’s always good to reinforce these things … it reinforces you to stick with what you know is best’* (School 3, mother).

#### Children trying new foods

Many children excitedly reported trying new foods as part of the intervention; *‘I never tried Weetabix with strawberries and bananas on it; it tastes really nice, now I eat it’* (School 5, child), although not all reported enjoying them; *‘I tried a blueberry but I didn’t like it’* (School 2, child). This exposure to healthy foods was an aspect of the programme that parents especially liked, a number of whom recounted children trying foods at the Cooking Workshops that they wouldn’t try at home. Equally, some parents reported that, since the intervention, children were more willing to try new foods in the home environment; *‘she’s willing to try more fruits and vegetables, that’s what I’m pleased with probably more, before she was quite picky with what she’d have, but now she is willing to try new things’* (School 7, mother). One parent, whose child was not keen to try any new foods at the Cooking Workshops, was still happy that children had been given these opportunities, and saw it as a positive learning experience; *‘unfortunately my son’s such a fussy eater, even though we tried, he wouldn’t try anything, I ended up having to try all the food [laugh] and he just wouldn’t even attempt it, but you know, he has learnt what is good and what is bad’* (School 6, mother).

#### Implementing change in the home

Following the intervention programme, some parents reported observing changes at home in terms of children’s interest in, and awareness of, healthy lifestyles; *‘he talks about his food more, about healthy eating and he tries to eat healthier’* (School 6, mother). A number of parents also reported children’s behaviour changes as a result of the intervention; *‘My son has made the change from more fizzy drinks and juice to water and he actually knows how much he needs to be consuming a day’* (School 5, mother).

Some parents reported making behavioural changes at home as a result of the intervention; *‘there’s definitely been a significant change …I’m very pleased to say that it* [WAVES] *has made a big difference in our household as a whole’* (School 5, mother). A number of parents made the connection between their children trying new foods within the intervention programme and behaviour change within the home environment; *‘she eat yoghurt, banana, fruits in the morning, before she never had that, before she liked toast and jam, all of us like it… now no more, don’t even buy them’* (School 9, mother).

Conversely, a small number of parents (from schools in more affluent areas) felt that the intervention programme had had no effect on their family as they were already leading healthy lifestyles; *‘it’s nothing different to what I would do at home if I’m being brutally honest.… it’s not going to have any impact or make any difference to me’* (School 8, mother).

#### Parental empowerment

A key emergent theme was that parents felt healthy lifestyle promotion at school (through the WAVES study activities) helped support and empower them to make changes at home. Parents recognised the powerful position of the teacher in conveying key healthy lifestyle messages to children. This was thought to a) be useful in promoting consistency between school and home, and b) have more of an effect than when the messages came from parents; *‘it’s good to have it reinforced I think from somebody other than your parents, sometimes if your teacher says it, it’s true!’* (School 8, father). As a result of the intervention, some parents felt empowered and supported in promoting healthy behaviours at home; ‘*It’s made it easy at home to say no fizzy drinks without making a fuss explaining’* (School 10, father).

#### Role modelling

Parents and children enthusiastically discussed the importance of healthy role modelling within the WAVES study intervention programme. For example, children were animated when discussing working with the Villa Vitality chef and football coaches, and one mother talked about the positive influence that the visits to the football club had on her son; *‘who wouldn’t want to be like a footballer?… they’re their role model and this is what they’re eating and this is the exercise they’re doing, what child’s not going to want to copy them?’* (School 10, mother). It was clear that children also viewed their teachers as role models, and particularly enjoyed it when teachers participated in the physical activities; *‘it’s good exercise for you and I like it when* [teacher’s name] *does it’* (School 4, child).

#### Children as agents of change in the home

From parental reports, it emerged that some children were helping to affect changes within the home environment, by encouraging parents to change their habits; *‘my son… he actually does have an issue with what I put in his lunchbox, you know, and it’s like ‘oh don’t give me a croissant all the time or don’t give me this all the time mum, you know, it’s not good’ so he’s made me think about it instead of just rushing around trying to get everything in there and get him off to school, it’s made me think twice about what I actually do put in his lunchbox’* (School 5, mother). Some parents viewed this positively as a role reversal; ‘*all them years of nag, nag, nag, nag ‘that’s not good for you, that’s not good for you’ but as soon as they do it in school ‘you can’t put sugar on my [cereal]…’* (School 10, mother).

### Sustainability

#### Sustainability of messages

Opinion differed on the sustainability of messages received through the intervention; some parents thought that the one-year intervention could have a long-term impact; *‘hopefully there’s enough embedded in them now that it’ll stay with them, you know, when they get older’* (School 3, mother), whilst others questioned the sustainability of effects. For example, in one focus group, parents discussed one of the Villa Vitality challenges (‘Eat 5 a day’) which involved children recording what fruits and vegetables they ate each day for one week. Whilst noting a positive impact in terms of children’s awareness and behaviour whilst undertaking the challenge, a longer-term effect was more questionable once the novelty of the intervention had passed; *‘…obviously once they’ve sort of had a few weeks of it, it just sort of disappears back into what they were sort of doing’* (School 1, mother).

#### Sustainability of school-based programmes

Parents acknowledged that school-based healthy lifestyle programmes should not be ‘one-offs’, and there was a need to re-visit the key messages. Some parents discussed how healthy lifestyles should be an important part of the curriculum in every year group; ‘*I think they should start at nursery and build themselves up as they go to year six’* (School 10, father). In addition, some parents expressed concerns about the transition from primary to secondary school in terms of healthy lifestyle promotion; ‘*once you get to secondary school it’s all about choice’* (School 10, mother).

### Responsibilities for obesity prevention

#### Role of parents

Although the focus of the group discussions was the WAVES study intervention, participants also considered the important role of parents in preventing obesity. All parents, whilst supportive of the intervention, felt they held the main responsibility for preventing obesity in their children; *‘at the end of the day you’re the parent, you’ve got to instil most of that into your children’* (School 1, mother) and recognised the need to set a good example; *‘as parents, you know, we’re role models’* (School 10, father). However, a number of barriers were discussed by children and parents that sometimes interfered with parents’ ability to deliver their responsibility. These barriers were raised in discussions about the ‘signposting sheets’, and could be seen as barriers to the effectiveness of the intervention, including the perceived high cost of healthy foods and activities, lack of local activities, limited space at home, siblings’ vying needs, the draw of sedentary activities, competing demands (e.g. religious practices) and lack of time; *‘it is difficult a lot of the time ‘cause I work, so by the time I’ve gone to work, get home from work it’s the timescale really, it’s bedtime before you know it’* (School 6, father).

#### Role of schools

Whilst accepting the main responsibility for obesity prevention, parents believed that schools also have an important role; *‘everybody has to encourage good eating and the schools have to be involved’* (School 8, mother). Parents felt that health promotion in schools, such as that delivered through the WAVES study, offered vital lessons to children to support their future health; *‘you’re thinking about the future, if you start healthy at this young age then obviously you don’t get overweight and all these diseases, diabetes, heart disease, they all arise from overweight’* (School 10, father).

*‘I think schools have to do it* [health promotion]*, it’s important isn’t it because as a society we’re not doing particularly well at eating healthily, sadly, so yeah it’s good if it can be taught and they can take something on board while they’re at school’* (School 3, mother).

Parents and children supported the inclusion of extra nutrition education and physical activities within school time, particularly those who struggled to find time for physical activity outside of school; *‘we’re Muslims, he goes to mosque as well, so he doesn’t really get much time in the evening to play about, so it’s good while they’re in school instead’* (School 6, mother). One child in particular discussed how the extra physical activity at school was important to her as she didn’t get many opportunities to be active at home; ‘*there’s not a lot of room in my house so I can’t do it* [physical activity]*…and my mum and dad said I’m not allowed to run around’* (School 7, child).

Furthermore, some parents and children perceived the additional physical activity undertaken through the WAVES study intervention to have a positive impact on concentration and learning; ‘*I think it makes them more active, more alert as well especially first thing in the morning, more able to learn and things… it’s very beneficial’* (School 9, mother).

*‘Because I’ve done my exercise I can think harder and try’* (School 6, child).

A few parents, whilst supportive, were aware of difficulties that schools faced with fitting in additional activities. There was a perceived hierarchy of activities that schools should deliver, with academic learning being relatively more important. For example, in relation to fitting extra physical activity into the school day, one parent stated: *‘it depends on whether it’s going to affect the rest of the academic things like their writing and spelling’* (School 6, mother).

Additionally, some parents expressed concerns about whether focusing on healthy lifestyles could promote eating disorders; *‘we don’t want plenty anorexics about, because even now we’ve got children in Year 1* [5-6 years old] *telling each other that they’re fat or I’m thin or I’m this or I’m that. So we need to be careful about diet, exercise and healthy eating’* (School 10, mother).

#### Schools in partnership with parents

Parents discussed working in partnership with schools to promote healthy lifestyles to children. They appreciated the opportunities presented by the WAVES study to be involved in their children’s learning, and saw this as a way of reinforcing messages learnt at school within the home environment; *‘you get a foresight into what they’re doing… you know what’s happening and also what you can do to make it better, or add to it’* (School 1, mother).

## Discussion

In this qualitative study, we found that parents and children value healthy lifestyle interventions delivered through schools, and report changes in knowledge, skills and family lifestyle behaviour as a result. There were concerns that changes in behaviour would not be sustained longer term. Several practical barriers to behaviour change, which could reduce intervention effects, were also discussed.

Parental involvement in health promotion interventions for children has been identified as an important factor in improving intervention effects [3]. Findings from this study suggest that such involvement improves parental knowledge and facilitates consistency of messages between school and home. A key theme was that intervention delivery through school, with teachers as role models and authoritative messengers, leads to a sense of empowerment for parents as they feel supported by schools in their attempts to promote healthy lifestyles for their children. Data from teacher interviews undertaken as part of the WAVES study process evaluation indicated that teachers were generally not in a position to assess the impact of the intervention on behaviour change [17], which may result in their underestimation of the positive effect of intervention delivery. Creating a feedback mechanism, to make teachers aware of intervention impact, may help motivate them to more consistently promote healthy lifestyles. In addition to parental empowerment and teacher influence, there was indication that children themselves were instrumental in influencing parents to implement lifestyles changes at home. This promising finding is similar to a recent study showing that empowering primary school children to educate their families was effective in lowering salt intake [23].

Food neophobia (a reluctance to try new foods) is believed to peak at the age of six years [24], and research suggests that novel food needs to be presented in a positive light, including highlighting the fun of preparing or cooking the food [24]. Willingness to try new foods has also been shown to increase when more people around the child consume the food [25]. We describe how the practical cooking aspects of the intervention, including preparation and trying of new foods by children (aged 6-7 years) alongside their classmates, parents and teachers, facilitated many to try new, healthy foods. This aspect of the intervention may have been successful in behaviour change which was translated to the home environment.

Although behaviour change theory was not explicitly used in the development of the WAVES study intervention, the empirical data from this study resonate with the framework set out in the Behaviour Change Wheel [26]. This has at its centre the COM-B model which describes three conditions necessary for behaviour change to occur; Capability, Opportunity and Motivation. This conceptual model can be used to theoretically explain the reported lifestyle changes resulting from the intervention. For example, improved skills in physical activity and nutrition (physical capability) alongside the empowerment of parents to implement changes with their children (psychological capability leading to increased motivation); the normalisation of healthy lifestyle behaviours, both in and out of school, e.g. at the football club (reflective motivation); positive role modelling from teachers and at the football club (automatic motivation), and the intervention programme providing occasions to promote and enact healthy lifestyle behaviours with children and families (physical and social opportunity). If capability, opportunity and motivation of children and parents, as well as schools and their staff, are addressed in future interventions, they may be more likely to result in behaviour change within families.

Parents and children in this study reported various barriers to behaviour change, many of which were also recognised by the teachers [17], and are consistent with findings from previous studies [11, 27]. This study also revealed a differential intervention impact on individual families, with some parents and children reporting significant behavioural changes, and others, despite appreciating the intervention as valuable education for children, reporting no impact as they considered themselves to be already leading healthy lifestyles. In considering the differential impact that the intervention might have had in different strata of the population, we posit that disparities observed could possibly be explained by the socio-economic circumstances of families, as our observations were that the parents who reported higher knowledge and existing healthier practices at home tended to be from schools serving areas of higher socioeconomic status. We propose that an important factor in this apparent potential of the WAVES study to affect positive lifestyle changes among families with poorer prior healthy lifestyle knowledge (which in this study tended to be amongst the participants from more deprived communities) was that the intervention targeted simple and achievable behaviour change. This variable impact, depending on family circumstances, resonates with some previous health behaviour change intervention research that showed greater effects amongst populations from lower, compared to higher socio-economic backgrounds [28, 29]. Although all schools have an important role to play in the promotion of healthy lifestyles, the level of involvement required is likely to vary depending on the circumstances of, and the challenges faced by, the families of the children who attend. Schools, and those developing school-based healthy lifestyle interventions, need to be sensitive to barriers faced by families, and consider the context of the home and local environment when designing programmes. Different families will have distinct capabilities, opportunities and motivations, depending on their social, cultural and economic circumstances. Tailoring programmes to suit local needs has been reported as an important approach for maximising parental compliance [30]. Our study supports this and suggests that future childhood obesity prevention intervention programmes need to incorporate a degree of flexibility to enable adaptation to individual school and family circumstances.

While participants perceived school-time as an important opportunity for children to be physically active, suggestions that physical activity had increased outside of the school setting were scarce. Equally, there were no reports of any positive impact of the physical activity ‘signposting sheets’. The fact that the physical activity component of the intervention was delivered only to children within school, with no parental involvement, combined with the barriers reported by participants when discussing the ‘signposting sheets’ (for example, high cost or lack of local activities, vying needs of siblings, lack of time, competing demands, and the draw of sedentary activities), suggest that the intervention is unlikely to have promoted physical activity outside of school.

Although the emphasis of the WAVES study intervention programme was encouragement of lifestyle behaviours to help children stay healthy, some parents discussed the possibility of a negative impact on children’s perception of body image and risk of developing eating disorders. The Cochrane review of interventions for preventing obesity in children [3] considered the potential harm of such interventions, and although few trials have considered this, none have reported any risk of eating disorders or other harms. It has been suggested, however, that programmes could simultaneously prevent eating disorders and obesity based on the idea that they have common risk factors [31]. In such a programme, the focus would be on health and behaviour change, regardless of weight status, alongside the promotion of positive body image and the acceptance of the diversity of body shapes and sizes [32].

The reports of positive behaviour change resulting from the intervention are encouraging, supporting the promotion of healthy lifestyles through schools. However, sustainability of the impact was a concern for parents. Whether children would retain the acquired knowledge, and have a continued motivation to implement it once the intervention ended, echoes concerns reported by teachers [17], suggesting that healthy lifestyle messages needed to be re-visited and embedded within the school curriculum. This issue of sustaining impact over time, and the need to embed effective interventions into standard practice has been raised previously [3]. Incorporating successful components of the WAVES study intervention programme into a ‘whole school approach’, advocated by the Health Promoting School model [5], would help improve its sustainability.

### Limitations

Focus groups were held in purposively sampled schools, and this study represents the views of those parents and children from the selected schools who agreed to participate. These participants may have been more interested in the topic of healthy lifestyles and therefore more motivated to attend a focus group. Parents and children who declined participation, as well as those from schools not selected for this study, may have offered different perspectives. With the exception of one school (School 10), the response rate from parents was quite low. Through an analysis of field notes taken during focus group discussion, we were able to consider group dynamics, both between participants, and between participants and researchers. Some of the focus groups had small numbers of participants (e.g. 2-3 participants), leading to (in a minority of groups) a reduced level of interaction between group members and limited exploration of shared perspectives. However, in most of the groups, good participant interactions were evident as they worked together to describe their experiences.

The fact that the researchers had some knowledge of participating schools and had previously met some of the participants on school visits as part of the WAVES study may have affected participant responses (e.g. social desirability bias). There may also have been a risk of bias in data interpretation (e.g. researcher pre-conceived ideas about schools or participants based on prior knowledge and experience).

Of the 30 parent participants, only five were male, and they were interviewed alongside female participants. Fathers’ views are therefore under-represented in this study. This gender bias is similar to other studies, and is likely a reflection of society, with mothers being the primary carers of children [14, 33, 34]. However, when the views of participating fathers were compared to those of mothers, the authors found no clear differences in opinion between male and female participants. Despite these limitations, the number of participants from a diverse range of schools enables tentative conclusions to be drawn about parent and child opinions of school-based obesity prevention programmes.

## Conclusions

This qualitative study enabled us to better understand aspects of the WAVES study intervention programme that have the potential to initiate positive behaviour changes in families, and indicated that a combination of pathways influenced such changes. Pathways included: increasing capability through improving knowledge and skills of children and parents; increasing motivation through parental empowerment and role modelling; and the direct provision of opportunities to lead healthier lifestyles. Strategies to sustain behaviour changes, and the school role in supporting these, are important considerations.

## Abbreviations

WAVES: West Midlands ActiVe lifestyle and healthy Eating in School children

## References

[1] World Health Organisation (2004). Global strategy on diet, physical activity and health.

[2] Lobstein T, Baur L, Uauy R (2004). Obesity in children and young people: a crisis in public health. Obes Rev.

[3] Waters E, de Silva SA, Hall B, Brown T, Campbell KJ, Gao Y (2011). Interventions for preventing obesity in children (review). Cochrane Collaboration.

[4] Clarke J, Fletcher B, Lancashire E, Pallan MJ, Adab P (2013). The views of stakeholders on the role of the primary school in preventing childhood obesity: A qualitative systematic review. Obes Rev.

[5] World Health Organisation. What is a health promoting school? 2013. Available at: http://who.int/school_youth_health/gshi/hps/en/index.html. Accessed 20 October 2015.

[6] Pawson R, Tilley N (1997). Realistic evaluation.

[7] Kingry MJ, Tiedje L, Friedman LL (1990). Focus groups: a research technique for nursing. Nurs Res.

[8] Moore GF, Audrey S, Barker M, Bond L, Bonell C, Hardeman W (2015). Process evaluation of complex interventions: Medical Research Council guidance. BMJ.

[9] Adab P, Pallan MJ, Lancashire ER, Hemming K, Frew E, Griffin T (2015). A cluster-randomised controlled trial to assess the effectiveness and cost-effectiveness of a childhood obesity prevention programme delivered through schools, targeting 6–7 year old children: the WAVES study protocol. BMC Public Health.

[10] Bucher Della Torre SB, Akré C, Suris JC (2010). Obesity prevention opinions of school stakeholders: a qualitative study. J Sch Health.

[11] Hesketh K, Waters E, Green J, Salmon L, Williams J (2005). Healthy eating, activity and obesity prevention: a qualitative study of parent and child perceptions in Australia. Health Promot Int.

[12] Pallan M, Parry J, Cheng KK, Adab P (2013). Development of a childhood obesity prevention programme with a focus on UK South Asian communities. Prev Med.

[13] Patino‐Fernandez AM, Hernandez J, Villa M, Delamater A (2013). School‐based health promotion intervention: parent and school staff perspectives. J Sch Health.

[14] Rawlins E, Baker G, Maynard M, Harding S (2013). Perceptions of healthy eating and physical activity in an ethnically diverse sample of young children and their parents: the DEAL prevention of obesity study. J Human Nutr Diet.

[15] Moore G, Audrey S, Barker M, Bond L, Bonell C, Cooper C (2014). Process evaluation in complex public health intervention studies: the need for guidance. J Epidemiol Community Health.

[16] Griffin TL, Pallan MJ, Clarke JL, Lancashire ER, Lyon A, Parry JM (2014). Process evaluation design in a cluster randomised controlled childhood obesity prevention trial: the WAVES study. Int J Behav Nutr Phys Act.

[17] Griffin TL, Clarke JL, Lancashire ER, Pallan MJ, Passmore S, Adab P. Teacher experiences of delivering an obesity prevention programme (The WAVES study intervention) in a primary school setting. Health Educ J. 2015;74(6):655-67.

[18] Elliott R, Timulak L, Miles J, Gilbert P (2005). Descriptive and interpretive approaches to qualitative research. A handbook of research methods for clinical and health psychology.

[19] Stewart DW, Shamdasani PN (2014). Focus groups: Theory and practice.

[20] Morgan M, Gibbs S, Maxwell K, Britten N (2002). Hearing children’s voices: methodological issues in conducting focus groups with children aged 7-11 years. Qual Res.

[21] Ritchie J, Spencer L, Bryman A, Burgess R (1993). Qualitative data analysis for applied policy research. Analysing Qualitative Data.

[22] Gale NK, Heath G, Cameron E, Rashid S, Redwood S (2013). Using the framework method for the analysis of qualitative data in multi-disciplinary health research. BMC Med Res Methodol.

[23] He FJ, Wu Y, Ma J, Feng X, Wang H, Zhang J (2013). A school-based education programme to reduce salt intake in children and their families (School-EduSalt): protocol of a cluster randomised controlled trial. BMJ Open.

[24] Dovey TM, Staples PA, Gibson EL, Halford JC (2008). Food neophobia and ‘picky/fussy’eating in children: a review. Appetite.

[25] Birch LL (1980). Effects of peer models’ food choices and eating behaviors on preschoolers’ food preferences. Child Dev.

[26] Michie S, van Stralen MM, West R (2011). The behaviour change wheel: a new method for characterising and designing behaviour change interventions. Implement Sci.

[27] Eyre ELJ, Duncan MJ, Birch SL, Cox V (2013). Environmental and school influences on physical activity in South Asian children from low socio-economic backgrounds: a qualitative study. J Child Health Care.

[28] Brown J, Michie S, Geraghty AW, Yardley L, Gardner B, Shahab L (2014). Internet-based intervention for smoking cessation (StopAdvisor) in people with low and high socioeconomic status: a randomised controlled trial. Lancet Respir Med.

[29] Gulliford M, Charlton J, Bhattaraj N, Rudisill C (2014). Social and material deprivation and the cost-effectiveness of an intervention to promote physical activity: cohort study and Markov model. J Public Health (Oxford).

[30] Sonneville KR, La Pelle N, Taveras EM, Gillman MW, Prosser LA (2009). Economic and other barriers to adopting recommendations to prevent childhood obesity: results of a focus group study with parents. BMC Pediatr.

[31] Sánchez-Carracedo D, Neumark-Sztainer D, López-Guimera G (2012). Integrated prevention of obesity and eating disorders: barriers, developments and opportunities. Public Health Nutr.

[32] O'Dea JA (2005). Prevention of child obesity: ‘First, do no harm’. Health Educ Res.

[33] Pocock M, Trivedi D, Wills W, Bunn F, Magnusson J (2009). Parental perceptions regarding healthy behaviours for preventing overweight and obesity in young children: a systematic review of qualitative studies. Obes Rev.

[34] Stenhammar C, Wells M, Åhman A, Wettergren B, Edlund B, Sarkadi A (2012). ‘Children are exposed to temptation all the time’–parents’ lifestyle‐related discussions in focus groups. Acta Paediatr.

